# Risk factors for incident hyperuricemia during mid-adulthood in African American and White men and women enrolled in the ARIC cohort study

**DOI:** 10.1186/1471-2474-14-347

**Published:** 2013-12-11

**Authors:** Mara A McAdams-DeMarco, Andrew Law, Janet W Maynard, Josef Coresh, Alan N Baer

**Affiliations:** 1 Department of Epidemiology, Johns Hopkins University Bloomberg School of Public Health, 2024 E. Monument St, Suite B-319, Baltimore, MD, USA; 2 Department of Surgery, Johns Hopkins University School of Medicine, Baltimore, MD, USA; 3 Division of Rheumatology, Johns Hopkins University School of Medicine, Baltimore, MD, USA; 4 Current affiliation: United States Food and Drug Administration, Baltimore, MD, USA

**Keywords:** Hyperuricemia, Urate, Epidemiology

## Abstract

**Background:**

Increased serum urate levels are associated with poor outcomes including but not limited to gout. It is unclear whether serum urate levels are the sole predictor of incident hyperuricemia or whether demographic and clinical risk factors also predict the development of hyperuricemia. The goal of this study was to identify risk factors for incident hyperuricemia over 9 years in a population-based study, ARIC.

**Methods:**

ARIC recruited individuals from 4 US communities; 8,342 participants who had urate levels <7.0 mg/dL were included in this analysis. Risk factors (including baseline, 3-year, and change in urate level over 3 years) for 9-year incident hyperuricemia (urate level of **>**7.0 g/dL) were identified using an AIC-based selection approach in a modified Poisson regression model.

**Results:**

The 9-year cumulative incidence of hyperuricemia was 4%; men = 5%; women = 3%; African Americans = 6% and whites = 3%. The adjusted model included 9 predictors for incident hyperuricemia over 9 years: male sex (RR = 1.73 95% CI: 1.36-2.21), African-American race (RR = 1.79 95% CI: 1.37-2.33), smoking (RR = 1.27, 95% CI: 0.97-1.67), <HS education (RR = 1.27, 95% CI: 0.99-1.63), hypertension (RR = 1.65, 95% CI: 1.30-2.09), CHD (RR = 1.57, 95% CI: 0.99-2.50), obesity (class I RR = 2.37, 95% CI: 1.65-3.41 and ≥ class II RR = 3.47, 95% CI: 2.33-5.18), eGFR < 60 (RR = 2.85, 95% CI: 1.62-5.01) and triglycerides (Quartile 4 vs. Quartile 1: RR = 2.00, 95% CI: 1.38-2.89). In separate models, urate levels at baseline (RR 1 mg/dL increase = 2.33, 95% CI: 1.94-2.80) and 3 years after baseline (RR for a 1 mg/dL increase = 1.92, 95% CI: 1.78-2.07) were associated with incident hyperuricemia after accounting for demographic and clinical risk factors.

**Conclusion:**

Demographic and clinical risk factors that are routinely collected as part of regular medical care are jointly associated with the development of hyperuricemia.

## Background

Increased serum urate levels are associated with poor outcomes including but not limited to gout [1-11]. There are clear differences in serum urate levels by age, sex, and race. Young adult women are known to have lower serum urate levels than young adult men, [12] which is primarily attributable to sex hormone effects on renal urate transport [13]. The onset of menopause is associated with increased serum urate levels [13,14]. Additionally, young African Americans have lower serum urate levels than whites; although African American women are at a higher risk than white women of developing hyperuricemia [12]. Previous studies have identified individual risk factors for increased serum urate levels and incident hyperuricemia in US populations in addition to age, sex, and race: diet, alcohol intake, medication use, and chronic conditions [12,13,15-22].

In current clinical practice, patients with asymptomatic hyperuricemia are not treated. Emerging research suggests that treatment of asymptomatic hyperuricemia may reduce the risk of some adverse health outcomes, such as mortality and cardiovascular disease; [23-27] therefore, treatment of asymptomatic hyperuricemia is currently being discussed to prevent adverse health outcomes [28,29]. Better understanding of which patients are at risk of developing hyperuricemia may aid in clinical decision-making about treatment of asymptomatic hyperuricemia. However, hyperuricemia risk prediction has been limited because previously published studies were cross-sectional, [12,14,17,21,22] and thus, only correlates of prevalent serum urate were identified in these studies. These cross-sectional studies do not distinguish whether the clinical factors associated with serum urate level predated the onset of hyperuricemia or whether hyperuricemia led to the onset of these clinical factors. Prospective studies of both men and women without hyperuricemia that follow participants until they develop this outcome are needed for accurate risk prediction to inform clinical decision-making. Additionally, these previous studies focused only on a single correlate of serum urate level [14,19-21]. However, optimum risk prediction for hyperuricemia requires the simultaneous assessment of the risk inferred by a combination of risk factors.

It is unclear what demographic and clinical risk factors contribute to the development of high uric acid levels and whether serum urate levels are the sole predictor of incident hyperuricemia or whether demographic and clinical risk factors also predict the development of hyperuricemia (>7.0 mg/dL). Therefore, we utilized a population-based cohort study that included prospective measurement of a range of demographic and clinical risk factors among African American and white, middle-aged men and women to identify risk factors for the development of hyperuricemia over 9 years.

## Methods

### Setting and participants

The Atherosclerosis Risk in Communities study (ARIC) is a prospective population-based cohort study of 15,792 individuals recruited from 4 US communities (Washington County, Maryland; Forsyth County, North Carolina; Jackson, Mississippi; and suburbs of Minneapolis, Minnesota). The Institutional Review Board of the participating institutions (Johns Hopkins University, University of North Carolina at Chapel Hill, Wake Forest Baptist Medical Center, University of Mississippi Medical Center, and University of Minnesota) approved the ARIC study protocol and study participants provided written informed consent. Participants aged 45 to 64 years were recruited to the cohort in 1987–1989. This cohort was established to study the natural history of atherosclerosis, and the study consisted of 1 baseline visit (visit 1) between 1987 and 1989 and 3 follow-up visits (visits 2, 3, and 4) administered 3 years apart. Details of the study design have been previously published [30].

We excluded participants with prevalent hyperuricemia at cohort entry, defined as a measured serum urate level >7.0 mg/dL at the baseline visit (n = 2,455) to ensure that those study participants who were included in the analysis were at risk of developing hyperuricemia during follow-up. We additionally excluded those who were missing baseline serum urate level (n = 77) because we were unsure whether or not they had hyperuricemia at baseline. This analysis was limited to participants who were Caucasian or African American; few participants reported other races (n = 48). Additionally, participants who did not have a subsequent urate measure were not included in this study (n = 4,288). Results were similar when multiple imputation was used to include participants with missing serum or plasma urate levels. Finally, we only included participants with complete ascertainment of demographic and clinical risk factors (n = 582 excluded). There were 8,342 participants included in our study; however, 8,211 participants were included in the analyses that adjusted for serum urate level at visit 2 due to the fact that 131 participants in the analytic cohort did not have a measure of serum urate level at visit 2. Participants who were excluded from this analysis did not differ on sex or age. However, they were more likely to be African-American (37% vs. 19%). Additionally, participants with missing plasma urate at visit 4 differed from those with available plasma urate based on baseline smoking status (39% vs. 23%), hypertension (38% vs. 26%), diabetes (17% vs. 8%), CHD (7% vs. 3%), and obesity (27% vs. 22%).

### Hyperuricemia

Serum urate concentrations were measured with the uricase method at visit 1 and 2 in mg/dL [31]. The reliability coefficient of serum urate was 0.91, and the coefficient of variation was 7.2% in a sample of 40 individuals with repeated measures taken at least 1 week apart [32]. At visit 4 plasma urate level was measured in mg/dL and hyperuricemia was defined as a plasma urate level **>**7.0 mg/dL at this visit. Participants who were free of hyperuricemia at visit 1 (by study design) and who had hyperuricemia at visit 4 were considered to have 9-year incident hyperuricemia.

### Demographic and clinical risk factors

All demographic and clinical risk factors were assessed at baseline and included: sex, age (in years), race (white or African American), smoking status, education (less than high school or high school and higher), diabetes, hypertension (>140/90 mm Hg or use of an anti-hypertensive treatment), diuretic use, coronary heart disease (CHD), total/HDL cholesterol, triglycerides level (mg/dL, categorized as quartiles), congestive heart failure (use of a medication for heart failure or fulfillment of the Gothenburg Criteria), body mass index (BMI, kg/m^2^), early adult obesity (from self-reported weight at age 25), weight change from age 25 to baseline, waist-to-hip ratio, alcohol intake (grams/week), animal protein/fat intake (grams/day), and menopausal status (self-reported for women pre- and peri- vs. post-menopausal). Glomerular filtration rate (eGFR) was estimated by using the CKD-Epi equation [33] and categorized as ≥90, 60–90, or <60 ml/min/1.73 m^2^. Categories were chosen to reduce residual confounding. All risk factors were categorized based on empirical categorizations to reflect the distribution in the cohort.

Additionally, serum urate levels at visits 1 and 2 were considered predictors of the future development of hyperuricemia. Baseline serum urate level, follow-up serum urate level (measured 3-years after baseline), and 3-year change in serum urate level were considered predictors of hyperuricemia.

### Analysis

First, the mean and standard deviation (SD) as well as the prevalence of the covariates were calculated and compared by incident hyperuricemia. The mean of continuous variables in those with incident hyperuricemia was compared with the mean of those who did not develop hyperuricemia using a *t*-test; the prevalence of categorical factors was compared using the Fisher’s exact tests. For categorical factors with more than 2 levels, a test for trend was conducted using a nominal value for each category.

Using modified Poisson regression, [34] the relative risk (RR) of 9-year incident hyperuricemia was calculated. All potential demographic and clinical risk factors, except serum urate measures, were considered in the initial model. We used an Akaike’s Information Criterion (AIC)-based selection criteria to create a final model of predictors of 9-year incident hyperuricemia. Then, baseline serum urate level, 3-year serum urate level, and 3-year change in serum urate level were included as a predictor of incident hyperuricemia in separate models that were adjusted for demographic and clinical risk factors. In sensitivity analysis, the threshold for serum urate level was set at 6.8 mg/dL.

## Results

### Study cohort characteristics

There were 8,342 participants included in our study; 63% were female and 19% were African-American. The 9-year cumulative incidence of hyperuricemia was 4% and subgroup cumulative incidence rates were: 5% for men; 3% for women; 6% for African Americans and 3% for whites.

Cohort demographic and clinical risk factors differed by incident hyperuricemia status (Table 1). Overall, participants who developed hyperuricemia were more likely to be male (49% vs. 37%, p-value < 0.001), and African American (33% vs. 18%, p-value < 0.001), and were less likely to have a high school education or higher (73% vs. 83%, p-value < 0.001). Furthermore, participants who developed hyperuricemia were more likely to have chronic conditions at baseline: hypertension (44% vs. 24%, p-value < 0.001), obesity (BMI ≥ 30 kg/m^2^; 37% vs. 21%, p-value < 0.001), diabetes (11% vs. 8%, p-value < 0.05), and CHD (6% vs. 3%, p-value < 0.05). Participants with incident hyperuricemia were more likely to have an eGFR <60 ml/min/1.73 m^2^ (4% vs. 1%) or 60–90 ml/min/1.73 m^2^ (40% vs. 39%; p-value for trend < 0.001) than those without hyperuricemia. Among female participants, who were free of hyperuricemia by design, there was no difference in menopausal status at baseline and incidence of hyperuricemia, which may be the result of the exclusion of those with hyperuricemia at baseline (Table 1).

**Table 1:** Baseline risk factors by incident hyperuricemia in the ARIC cohort

	***No incident hyperuricemia***	***Incident hyperuricemia***
**Baseline risk factors**	***n = 7,908***	***n = 304***
**Male sex**	37	49**
**Age, years**	53.8 (6)	54.3 (5)
**African American race**	18	33**
**High school education or higher**	83	73**
**Pre-menopause**^**a,b**^	35	37
**Smoking status**^**a**^		
Current smoker	22	27
Former smoker	31	28
Never smoker	47	45
**Diuretic use**	12	19**
**Hypertension**	24	44**
**Total cholesterol, mmol/L**	6 (1)	6 (1)
**HDL cholesterol**^**a**^		
< 1.03 mmol/L	20	26*
1.03 ≤ HDL ≤ 1.55	48	50
> 1.55	32	24
**Diabetes**	8	11*
**Coronary heart disease**	3	6*
**Congestive heart failure**	2	5*
**BMI, kg/m**^**2a**^		
BMI ≥ 35	6	14**
30 ≤ BMI < 35	15	23
25 ≤ BMI < 30	39	45
BMI < 25	40	18
**Early adult obesity**	3	7**
**Weight change from early adult, lb**	25 (26)	32 (30)**
**Waist-to-hip ratio, quartiles**^**a**^		
Waist-to-hip ratio ≥ 0.97	23	38**
0.92 ≤ Waist-to-hip ratio < 0.97	25	28
0.86 ≤ Waist -to-hip ratio < 0.92	26	22
0.86 ≤ Waist-to-hip ratio	26	12
**Categorical alcohol intake, grams/week**^**a**^		
Abstain	61	63*
0–100	27	24
>100	12	13
**Animal fat, grams/day**	35 (17)	37 (18)*
**Animal protein, grams/day**	53 (23)	53 (22)
**eGFR, ml/min/1.73 m**^**2a**^		
<60	1	4**
60–90	39	40
≥ 90	60	56
**Baseline serum urate level, mg/dL**	5.3 (1.0)	6.1 (0.78)**
**Serum urate level at 3 year follow-up, mg/dL**	5.5 (1.2)	7.0 (1.3)**
**3-year change in serum urate level, mg/dL**	0.2 (0.9)	0.9 (1.3)**

Mean and standard deviation (SD) for continuous variables and percent for categorical variables.

*p-value < 0.05 ; **p-value < 0.001.

^a^p-value for trend.

^b^The number of participants included in analysis was 4,551; men (n = 3,036) and women without known menopause status (n = 625) were excluded from this analysis.

Participants who developed hyperuricemia were more likely to have a higher serum urate at baseline, even though the level did not cross the threshold of hyperuricemia by design (6.1 vs. 5.3 mg/dL, p-value < 0.001). This trend persisted during follow-up (3 years after baseline) (7.0 vs. 5.5 mg/dL, p-value < 0.001). Furthermore, there was a greater change in serum urate levels over 3 years (0.87 vs. 0.19, p-value < 0.001) for those who developed hyperuricemia.

### Demographic and clinical risk factors for incident hyperuricemia

The final adjusted model included 9 demographic and clinical risk factors for incident hyperuricemia over 9 years (Table 2). Men were at 1.73-fold (95% CI: 1.36-2.21) increased risk of developing hyperuricemia and African-American participants were at 1.79-fold (95% CI: 1.37-2.33) increased risk. Additional participant demographics such as current smoker (RR = 1.27, 95% CI: 0.97-1.67) and less than a high school education (RR = 1.27, 95% CI: 0.99-1.63) were associated with an increased risk of developing hyperuricemia. Participants with hypertension were at 1.65-fold (95% CI: 1.30-2.09) increased risk of developing hyperuricemia and CHD was associated with an increased but not significant risk of hyperuricemia (RR = 1.57, 95% CI: 0.99-2.50). Additionally, BMI was a strong predictor of incident hyperuricemia; there was an increased risk of developing hyperuricemia for participants who were overweight (RR = 2.01, 95% CI: 1.46-2.78), or had 30 ≤ BMI < 35 kg/m^2^ (RR = 2.37, 95% CI: 1.65-3.41) and BMI ≥ 35 kg/m^2^ (RR = 3.47, 95% CI: 2.33-5.18). eGFR < 60 (RR = 2.85, 95% CI: 1.62-5.01) at baseline was associated with incident hyperuricemia. Finally, high triglyceride levels were a predictor of incident hyperuricemia (quartile 4 vs. quartile 1: RR = 2.00, 95% CI: 1.38-2.89). There were no differences in the association of smoking status, education, hypertension, BMI, or kidney function between men and women as well as between African Americans and white participants (all p-values for these interactions >0.05).

**Table 2:** Predictors of incident hyperuricemia over 9 years in the ARIC cohort

	**Model 1**^**a **^**RR (95% CI)**	**Model 2**^**a **^**RR (95% CI)**	**Model 3**^**b **^**RR (95% CI)**	**Model 4**^**b **^**RR (95% CI)**
**Risk factor**				
**Male sex**	1.73 (1.36, 2.21)	1.10 (0.85, 1.43)	1.02 (0.80, 1.31)	1.54 (1.21, 1.97)
**African American race**	1.79 (1.37, 2.33)	1.68 (1.29, 2.18)	1.50 (1.15, 1.94)	1.64 (1.26, 2.13)
**<High school education**	1.27 (0.99, 1.63)	1.29 (1.00, 1.65)	1.15 (0.89, 1.49)	1.23 (0.96, 1.58)
**Hypertension**	1.65 (1.3, 2.09)	1.47 (1.15, 1.87)	1.27 (0.99, 1.62)	1.44 (1.12, 1.84)
**Coronary heart disease**	1.57 (0.99, 2.50)	1.60 (1.00, 2.56)	1.48 (0.90, 2.43)	1.45 (0.89, 2.37)
**Smoking status**				
Current smoker	1.27 (0.97, 1.67)	1.29 (0.98, 1.69)	1.26 (0.96, 1.66)	1.27 (0.96, 1.66)
Former smoker	0.84 (0.64, 1.11)	0.81 (0.62, 1.07)	0.78 (0.60, 1.03)	0.83 (0.63, 1.09)
Never smoker	*Reference*	*Reference*	*Reference*	*Reference*
**Body mass index, kg/m**^**2**^				
BMI ≥ 35	3.47 (2.33, 5.18)	2.07 (1.37, 3.14)	2.18 (1.44, 3.31)	3.25 (2.16, 4.9)
30 ≤ BMI < 35	2.37 (1.65, 3.41)	1.76 (1.22, 2.54)	1.90 (1.31, 2.75)	2.37 (1.64, 3.40)
25 ≤ BMI < 30	2.01 (1.46, 2.78)	1.64 (1.19, 2.26)	1.79 (1.30, 2.46)	2.04 (1.48, 2.81)
BMI < 25	*Reference*	*Reference*	*Reference*	*Reference*
**eGFR, ml/min/1.73 m**^**2**^				
<60	2.85 (1.62, 5.01)	1.97 (1.12, 3.47)	2.05 (1.24, 3.39)	2.86 (1.70, 4.82)
60-90	1.12 (0.89, 1.42)	0.95 (0.75, 1.20)	1.05 (0.84, 1.32)	1.18 (0.94, 1.48)
≥ 90	*Reference*	*Reference*	*Reference*	*Reference*
**Triglyceride, mg/dL**				
Quartile 4: ≥ 157	2.00 (1.38, 2.89)	1.48 (1.03, 2.13)	1.44 (1.01, 2.04)	1.93 (1.35, 2.76)
Quartile 3: 110 -156	1.46 (1.01, 2.12)	1.20 (0.83, 1.73)	1.12 (0.79, 1.60)	1.41 (0.99, 2.02)
Quartile 2: 79 - 109	1.08 (0.73, 1.60)	0.96 (0.65, 1.41)	1.00 (0.68, 1.46)	1.12 (0.76, 1.65)
Quartile 1: ≤ 78	*Reference*	*Reference*	*Reference*	*Reference*
**Serum Urate Level at Visit1, mg/dL**		2.33 (1.94, 2.80)		
**Serum Urate Level at Visit2, mg/dL**			1.92 (1.78, 2.07)	
**Change in serum urate level (Visit 2-Visit 1), mg/dL**				1.60 (1.47, 1.74)

^a^The number of participants included in analysis was 8,342.

^b^The number of participants included in analysis was 8,211.

Note: Relative risks and 95% confidence intervals are reported. Each model includes all the risk factors with relative risks and 95% confidence intervals listed in the column. Hyperuricemia was defined as serum urate levels greater than 7.0 mg/dL.

### Serum urate level and incident hyperuricemia

Serum urate levels at baseline (RR for a 1 mg/dL increase =2.33, 95% CI: 1.94-2.80) and at visit 2 (RR for a 1 mg/dL increase =1.92, 95% CI: 1.78-2.07) were associated with the development of hyperuricemia after accounting for demographic and clinical risk factors (Table 2). Adjusting for baseline and follow-up (3 years after baseline) serum urate levels attenuated the associations of the demographic and clinical risk factors for hyperuricemia. Additionally, the 3-year change in serum urate level was associated with an increased risk of hyperuricemia, such that for every 1 mg/dL increase in serum urate, participants were 1.60-times (95% CI: 1.47-1.74) more likely to develop hyperuricemia after accounting for demographic and clinical risk factors. The association of serum urate level did not differ by sex (women RR = 2.49, 95% CI: 1.96-3.17; men RR = 2.41, 95% CI: 1.77-3.27; p-value for interaction = 0.98).

The results did not materially change when we defined incident hyperuricemia as a serum urate level >6.8 mg/dL. Additionally, the results were similar when the analysis was restricted to post-menopausal women (urate level at visit 1: RR = 2.36, 95% CI: 1.65-3.37; urate level at visit 2: RR = 1.96, 95% CI: 1.74-2.20; change in urate level RR = 1.73, 95% CI: 1.50-2.01).

## Discussion

The results from this large population-based cohort of middle-aged, African American and White, men and women followed over 9 years help define significant predictors of the onset of hyperuricemia. Although baseline serum urate level was a strong predictor of the incidence of hyperuricemia, it was not the sole factor. Other risk factors, including sex, race and chronic conditions, were important predictors of incident hyperuricemia above and beyond serum urate levels.

Our findings support and extend previous observations concerning risk factors for gout and hyperuricemia. Similar to prior studies, we determined that African Americans have an increased risk of incident hyperuricemia relative to whites. The effect was similar for African American men and women [35]. Congestive heart failure (RR = 1.67, 95% CI: 1.21-2.23) and diuretic use (RR = 3.32, 95% CI: 3.06-3.61) were associated with hyperuricemia among men with a high cardiovascular risk profile in the Multiple Risk Factor Interventional Trial [19]. However, the ARIC cohort is a low cardiovascular disease risk population and we did not identify these factors as predictors of incident hyperuricemia. Our study supports previous ones that identified modifiable risk factors as correlates of serum urate level [15,17,36]. In our study, BMI was a strong predictor, although alcohol intake was not independently associated with incident hyperuricemia. In contrast to previous cross sectional studies, [16] our multivariate analyses did not support an association of dietary factors with incident hyperuricemia in this cohort. One possible explanation for why the dietary factors were not associated with incident hyperuricemia is that by middle age, chronic conditions like obesity, hypertension, and kidney disease, are more likely to influence the development of hyperuricemia than the transient effects of dietary changes. Additionally, our measure of usual dietary intake may not fully capture the variation in purine-rich foods and thus, including these dietary factors may not improve our ability to predict who develops hyperuricemia. Finally, among women, we did not observe an association between menopausal status and incident hyperuricemia, as has been noted in other studies [13]. This finding may reflect the fact that our study population was middle-aged at enrollment and few female participants were pre-menopausal at the time of outcome ascertainment.

In current clinical practice, patients with asymptomatic hyperuricemia are not treated. This practice was strongly recommended in 1978 based on limited evidence at the time that hyperuricemia was associated with hypertension, atherosclerosis, cerebrovascular disease, as well as renal stones, and gouty nephropathy [37]. However, since 1978, many research studies have focused on the consequences of hyperuricemia providing strong evidence that links hyperuricemia directly to not only gout but other poor outcomes such as heart failure, atherosclerosis, endothelial dysfunction, sleep disordered breathing, diabetic nephropathy, metabolic syndrome, acute myocardial infarction, stroke, ischemic heart disease, hypertension, chronic kidney disease, acute kidney injury, and death [3-11,23,38-50]. Additionally, there is growing evidence that treatment of asymptomatic hyperuricemia improves health outcomes for patients. Treatment of asymptomatic hyperuricemia is currently being discussed to prevent adverse health outcomes [28,29].

Treatment of asymptomatic hyperuricemia has been found to improve health outcomes in patients. There is emerging evidence that reductions in serum urate levels have led to improved health including improvements in endothelial dysfunction, inflammation, and kidney function in asymptomatic patients [23-26] and cardiovascular events and mortality among those with prescribed allopurinol [27]. Evidence from clinical trials suggests that treatment of asymptomatic hyperuricemia controls essential hypertension [51]. In obese adolescents with prehypertension, urate lowering therapy reduced systolic BP by 10.2 mm Hg and diastolic BP by 9.0 mm Hg in treated patients compared with a rise of 1.7 mm Hg and 1.6 mm Hg in systolic and diastolic blood pressure for patients on placebo [52]. Therefore, urate lowering therapy reduces systemic vascular resistance [52]. Our findings may help guide clinical decision-making to help identify which patients are at highest risk of developing hyperuricemia based on patient characteristics, chronic conditions, and serum urate levels.

There are a few notable limitations to this study. First, we do not have measures of serum urate level prior to enrollment in ARIC. Therefore, we cannot be certain that incident hyperuricemia over follow-up is truly the first occurrence of hyperuricemia. Next, not all participants had a measure of serum urate level after baseline. There is limited evidence of differential missing plasma urate measures and thus, the potential for survival bias is minimal. There was no serum measure of sex hormones, which have been associated with serum urate level [53]. Therefore we were unable to assess the impact of sex hormones on the incidence of hyperuricemia. Furthermore, there were too few pre- or peri-menopausal women with hyperuricemia (n = 51) to identify risk factors in this subgroup. Additionally, there were no participants who had received kidney transplantation, a strong risk factor for hyperuricemia, and thus our results are not generalizable to this clinical population [54]. The strengths of this study include multiple measures of serum urate, which allows for the analysis of incident hyperuricemia rather than correlates of serum urate level. Furthermore, ARIC is a rich cohort with detailed data collection from African American and white, men and women.

## Conclusions

These study findings extend the previous research of correlates to serum urate level to the development of incident hyperuricemia. Our results suggest that serum urate levels alone do not predict incident hyperuricemia; patient characteristics and chronic conditions are also predictors of the development of hyperuricemia. The factors identified as predictors of incident hyperuricemia are routinely collected as part of regular medical and may be readily incorporated into risk prediction. Future work should establish the clinical effectiveness of predicting and treating incident hyperuricemia to prevent the onset of gout and other adverse outcomes.

## Competing interest

The authors declare that they have no competing interests.

## Authors’ contributions

MMD made substantial contributions to conception and design, acquisition of data, analysis and interpretation of data; and was involved in drafting the manuscript and revising it critically for important intellectual content. JM made substantial contributions to conception and design, interpretation of data, and was involved in revising the manuscript critically for important intellectual content. AL made substantial contributions to design, acquisition of data, analysis and interpretation of data, and was involved in drafting the manuscript and revising it critically for important intellectual content. JC made substantial contributions to conception and design, acquisition of data, analysis and interpretation of data, and was involved in drafting the manuscript or revising it critically for important intellectual content. AB made substantial contributions to conception and design, analysis and interpretation of data, and was involved in drafting the manuscript and revising it critically for important intellectual content. All authors gave final approval of the version to be published.

## Pre-publication history

The pre-publication history for this paper can be accessed here:

http://www.biomedcentral.com/1471-2474/14/347/prepub
